# Supplementary material for: Mussel larvae modify calcifying fluid carbonate chemistry to promote calcification
Source: Nat Commun. 2017 Nov 22;8:1709. doi: 10.1038/s41467-017-01806-8 (PMC5700083; doi:10.1038/s41467-017-01806-8)
Supplement: Supplementary file 1 — Supplementary Information [file 41467_2017_1806_MOESM1_ESM.pdf]

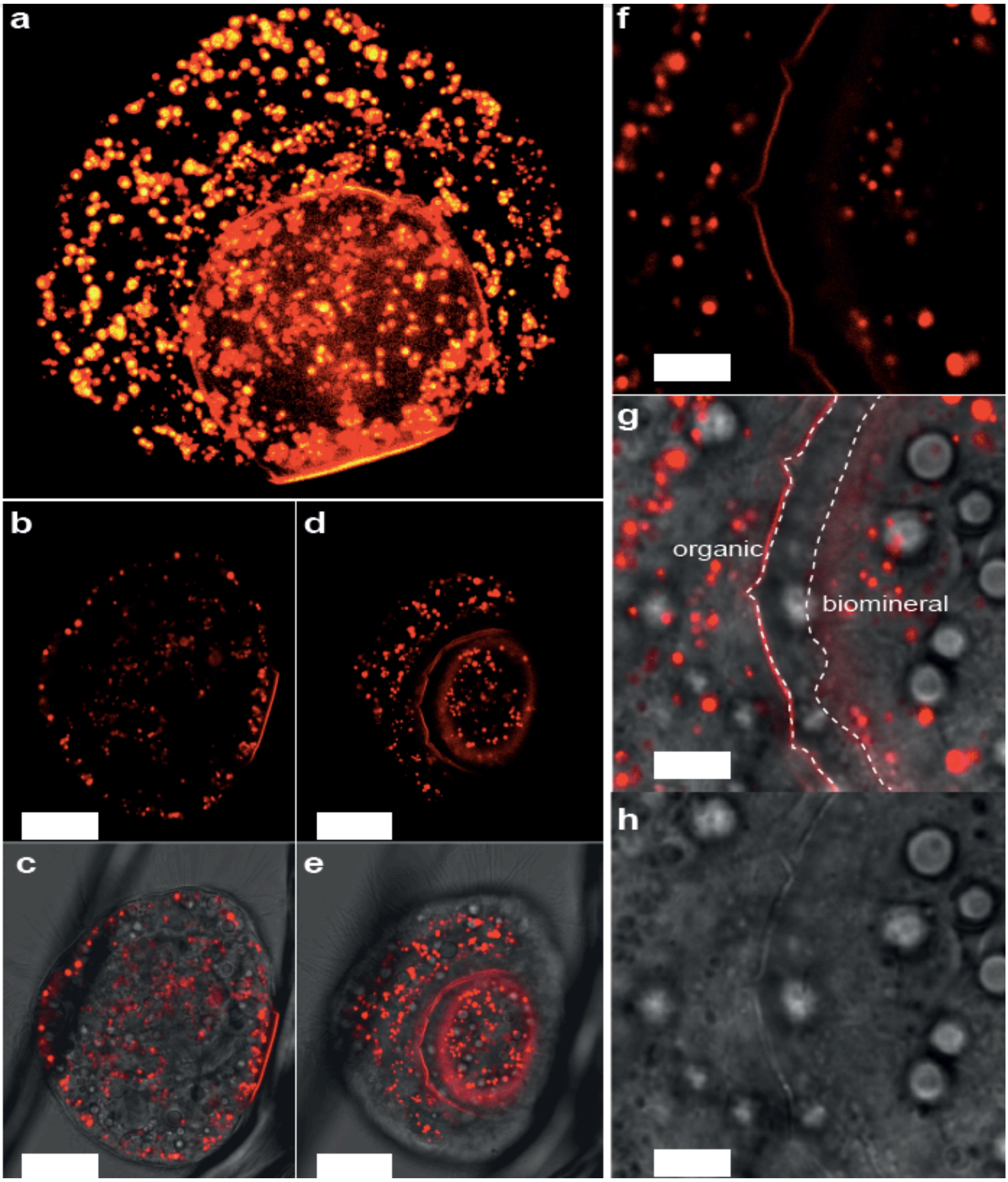

Supplementary Figure 1. Shell organic cover autofluorescence images. (**a**) Confocal projection of unstained 27hpf larva imaged at high laser intensity and using a long – pass filter (LP 505 nm) instead of the band – pass filter (BP 505-530 nm) used for calcein imaging. Autofluorescence of shell organic cover and of numerous vesicles is visible; (**b**) confocal section illustrating autofluorescence of organic portion of hinge. The hinge is not calcified at this point of time (see Fig. 1) (**c**) same larva, merged transmission and fluorescence images (**d**) same larva, different optical plane to illustrate organic shell cover; (**e**) same larva, merged transmission and fluorescence images (**f, g, h**) same larva, close – up of shell margin fluorescence (**f**), merged fluorescence and transmission images (**g**) and transmission light

image (**h**). Organic shell cover and organic shell matrix are fluorescent and illustrate the ca. 3-5 µm gap between growing edges of organic vs. biomineral phases. Scale bars: 20 µm (b,c,d,e), 5 µm (f,g,h).

Supplementary Table 1. Seawater carbonate chemistry parameters calculated from C_T and pH_{NBS} using CO2SYS. Carbonate chemistry measurements were obtained once for each fertilization / experimental run. The last three columns indicate, which measurements were conducted with the respective experimental runs (a: for Fig. 3 and 4 (microsensors), b: for Fig. 5 (shell length), c: for Fig. 5 (shell dissolution)).

Exp	Sal. (psu)	Temp. (°C)	Treat ment	C_T (µmol/ kg SW)	pH_{NBS}	pCO_2 (µatm)	A_T (µmol/kg SW)	CO_3^{2-} (µmol/kg SW)	Ω_{arag}	a	b	c
Exp	15.1	16.5	1	1915.83	8.19	445	2018.91	94.66	1.52	X		
3_1	15.1	16.5	2	1955.05	8.07	607	2026.11	74.44	1.19	X		
	15.1	16.5	3	1994.27	7.86	1020	2013.69	46.71	0.75	X		
	15.1	16.5	4	2042.31	7.73	1398	2032.53	35.68	0.57	X		
	15.1	16.5	5	2045.25	7.64	1721	2014.06	28.89	0.46	X		
Exp	15.0	16.5	1	2008.59	8.14	534	2098.89	88.09	1.41	X	X	
3_2	15.0	16.5	2	2033.09	8.04	687	2096.73	71.59	1.15	X	X	
	15.0	16.5	3	2055.64	7.86	1054	2074.63	47.85	0.77	X	X	
	15.0	16.5	4	2083.09	7.61	1879	2043.21	27.24	0.44	X	X	
	15.0	16.5	5	2088.97	7.40	3004	1990.86	16.56	0.27	X	X	
Exp	14.8	16.5	1	1903.7	8.12	528	1984.40	79.58	1.27	X	X	
3_3	14.8	16.5	2	1938.99	8.01	693	1992.87	63.74	1.02	X	X	
	14.8	16.5	3	1986.04	7.77	1229	1985.66	37.96	0.61	X	X	
	14.8	16.5	4	2010.55	7.66	1602	1984.70	29.68	0.48	X	X	
	14.8	16.5	5	2018.39	7.60	1842	1978.15	25.88	0.41	X	X	
Exp	13.8	16.5	1	1917.55	8.16	474	2004.39	84.45	1.35	X	X	
3_4	13.8	16.5	2	1956.65	8.09	572	2025.49	73.53	1.18	X	X	
	13.8	16.5	3	1982.23	7.81	1112	1986.16	39.46	0.63	X	X	
	13.8	16.5	4	2040.05	7.66	1616	2009.33	28.57	0.46	X	X	
	13.8	16.5	5	2038.88	7.59	1892	1991.36	24.22	0.39	X	X	
Exp	15.4	16.8	1	1903.70	8.14	500	1995.87	87.01	1.40		X	
3_5	15.4	16.8	2	1938.99	8.00	701	1995.66	65.44	1.05		X	
	15.4	16.8	3	1986.05	7.76	1250	1986.99	38.77	0.62		X	
	15.4	16.8	4	2010.55	7.65	1634	1985.46	30.23	0.49		X	
	15.4	16.8	5	2018.39	7.55	2064	1968.32	23.87	0.38		X	
Exp	15.2	16.8	1	1899.53	8.16	476	1996.34	90.14	1.45		X	
3_6	15.2	16.8	2	1953.97	8.03	666	2016.56	69.35	1.11		X	
	15.2	16.8	3	2001.23	7.78	1220	2004.86	40.15	0.64		X	
	15.2	16.8	4	2030.44	7.68	1555	2010.82	32.29	0.52		X	
	15.2	16.8	5	2056.19	7.58	1974	2011.90	25.86	0.42		X	
Exp	14.6	16.8	1	1909.79	8.14	507	1997.65	84.70	1.36			X
4_1	14.6	16.8	2	1945.37	7.99	735	1994.95	61.61	0.99			X
	14.6	16.8	3	2009.76	7.77	1264	2008.99	38.46	0.62			X
	14.6	16.8	4	2030.36	7.69	1533	2011.12	32.26	0.52			X
	14.6	16.8	5	2059.10	7.57	2038	2010.33	24.68	0.40			X
	14.6	16.8	6	2084.12	7.47	2577	2007.78	19.69	0.32			X

Supplementary Table 2. Compiled information related to regressions reported in Fig. 4 with N=4 experimental fertilizations and 4-5 treatment levels per fertilization. Regressions were conducted on treatment mean values (all 4 replicate fertilizations). Test for heterogeneity of slopes were conducted using slopes of regressions for individual fertilizations. SW = sea water, CS = calcifying space, x = pCO_2 in µatm.

Parameter	Compartment	Regression Equation	ANOVA	P-value	R^2	Heterogeneity of Slopes	Intercepts
pH_{NBS}	SW<br>CS	y = -0.0004x+8.3191<br>y = -0.0006x+8.5765	$F_{(1,17)}$=303.2<br>$F_{(1,17)}$=516.3	<0.001<br><0.001	0.94<br>0.96	**P=0.01, t=5.2, df=3**	**P=0.001, t=15.8, df=3**
ΔpH_{NBS}	CS-SW	y = -0.0001x+0.2574	$F_{(1,17)}$=139.1	<0.001	0.89		
$\Delta[H^+]$ nmol kg^{-1}	CS-SW	y = 0.84+4/(1+exp(-(x-1612)/-16.6))	$F_{(3,15)}$=10.7	<0.001	0.68		
$[CO_3^{2-}]$ µmol kg^{-1}	SW<br>CS	y = -43.51ln(x)+350.01<br>y = -48.51ln(x)+395.23	$F_{(1,17)}$=22.54<br>$F_{(1,17)}$=27.37	<0.001<br><0.001	0.89<br>0.86	P>0.05, t=0.3, df=3	P>0.05, t=2.1, df=3
$\Delta[CO_3^{2-}]$ µmol kg^{-1}			$F_{(1,17)}$=2.546	>0.05	0.13		
Log $\Omega_{aragonite}$	SW<br>CS	y = -0.001x+0.7236<br>y = -0.0013x+1.2994	$F_{(1,16)}$=263.2<br>$F_{(1,16)}$=468.3	<0.001<br><0.001	0.94<br>0.96	**P<0.05, t=3.7, df=3**	**P=0.004, t=8.0, df=3**
Δlog $\Omega_{aragonite}$	CS-SW	y = -0.0004x+0.6595	$F_{(1,16)}$=155.5	<0.001	0.90		
$[Ca^{2+}]$ mmol kg^{-1}	SW<br>CS		$F_{(1,17)}$=0.82<br>$F_{(1,17)}$=0.58	>0.05<br>>0.05	0.04<br>0.03		
$\Delta[Ca^{2+}]$ mmol kg^{-1}	CS-SW		$F_{(1,17)}$=0.20	>0.05	0.01		
