# Supplementary material for: Mussel larvae modify calcifying fluid carbonate chemistry to promote calcification
Source: Nat Commun. 2017 Nov 22;8:1709. doi: 10.1038/s41467-017-01806-8 (PMC5700083; doi:10.1038/s41467-017-01806-8)
Supplement: Supplementary file 3 — Description of Additional Supplementary Files [file 41467_2017_1806_MOESM3_ESM.pdf]

Description of Additional Supplementary Files

File Name: Supplementary Movie 1
Description: Swimming larvae under crossed polarized light. 24 degree post fertilization (dpf) larvae were filmed free swimming at 100x magnification in a small glass dish filled with a 3 mm deep filtered seawater (FSW) layer at 17°C using an inverted microscope (Leica DMi8). The video demonstrates that the entire shell is birefringent. Length of sequence: 22 seconds. Length of larvae: ca. 70 µm, shell length: ca. 30-40 µm.
